# A systematic review on hospital inefficiency in the Eastern Mediterranean Region: sources and solutions

**DOI:** 10.1186/s12913-019-4701-1

**Published:** 2019-11-12

**Authors:** Hamid Ravaghi, Mahnaz Afshari, Pavaneh Isfahani, Victoria D. Bélorgeot

**Affiliations:** 1 Department of Health Service Management, School of Health Management and Information Sciences, Iran University of Medical Sciences, Tehran, Iran; 2 School of Public Health, Zabol University of Medical Sciences, Zabol, Iran; 3 World Health Organization, Regional Office for the Eastern Mediterranean, Monazamet El Seha El Alamia Street, Extension of Abdel Razak El Sanhouri Street, Nasr City, Cairo, Egypt

**Keywords:** Efficiency, Hospitals, Eastern Mediterranean countries, Systematic review

## Abstract

**Background:**

Evaluating hospital efficiency is a process to optimize resource utilization and allocation. This is vital due to hospitals being the largest financial cost in a health system. To limit avoidable uses of hospital resources, it is important to identify the sources of hospital inefficiencies and to put in place measures towards their reduction and elimination. Thus, the purpose of this research is to examine the sources of hospital inefficiency in the Eastern Mediterranean Region, and existing strategies tackling this issue.

**Methods:**

In this study, the electronic databases MEDLINE (via PubMed), Web of Science, Embase, Google, Google Scholar, and reference lists of selected articles, were explored. Studies on inefficiency, sources of inefficiency, and strategies for inefficiency reduction in the Eastern Mediterranean region hospitals, published between January 1999 and May 2018, were identified. A total of 1466 articles were selected using the initial criteria. After further reviews based on the inclusion and exclusion criteria, 56 studies were eligible for this study. The chosen studies were conducted in Iran (*n* = 35), Saudi Arabia (*n* = 5), Tunisia (*n* = 5), Jordan (*n* = 4), Pakistan (*n* = 2), the United Arab Emirates, Palestine, Iraq, Oman, and Afghanistan (*n* = 1 each). These studies were analyzed using content analysis in MAXQDA 10.

**Results:**

The analysis showed that approximately 41% of studies used data envelopment analysis (DEA) to measure hospital efficiency. Sources of hospital inefficiency were divided into four categories for analysis: Hospital products and services, hospital workforce, hospital services delivery, and hospital system leakages.

**Conclusion:**

This study has revealed some sources of inefficiency in the Eastern Mediterranean Region hospitals. Inefficiencies are thought to originate from excess workforce, excess beds, inappropriate hospital sizes, inappropriate workforce composition, lack of workforce motivation, and inefficient use of health system inputs. It is suggested that health policymakers and managers use this evidence to develop appropriate strategies towards the reduction of hospital inefficiency.

## Background

Hospitals are an essential component of health systems, while also being the most costly. They account for 50–80% of total health expenditures [1]. Hospital costs continue to rise due to the development of new technologies. New diagnostic and therapeutic methods are implemented to combat the rising proportion of chronic diseases, the increasing demand for health services, and the subsequent medical errors [2]. This has become a primary challenge and concern for governments [3].

Hospitals in the Eastern Mediterranean Region (EMR) differ in size, proprietorship, assignment, and performance. The total number of hospital beds is estimated to be 740,000 and, except for Lebanon, the majority of hospital beds are in the public sector (80%), with the remaining in private for-profit (18%) and private not-for-profit (2%) hospitals. The range of hospital beds per 10,000 population vary from 3.9 to 32 in 22 countries in the EMR. Hospitals also vary widely in size, location (rural and urban), resources, specialization (general versus specialty hospitals) and organization, as well as their position in the health system (first-level hospitals, secondary care hospitals and large teaching institutions) [4]. A large proportion of hospitals are financed by the government, but out-of-pocket payments are rising due to limited public sector resources [5]. This leads to limited access to health services for vulnerable communities. Private hospitals in the EMR are usually small to medium size and located in capitals and other large cities. These hospitals are not the result of comprehensive health system planning, as such, they can also lead to inequity in access to healthcare. Most countries in the EMR have addressed inequalities by implementing reforms to increase productivity, transparency, and cost flexibility [5–7]. To facilitate this process and increase hospital efficiency, it is necessary to provide the healthcare sector with additional resources and management tools.

According to Farrell (1957), efficiency is defined as “the firm’s success to produce the maximum feasible amount of output from a given amount of input or producing a given amount of output using the minimum level of inputs where both the inputs and the outputs are correctly measured” [8]. Three different types of efficiency were defined by Farrell: technical efficiency, allocative efficiency, and economic efficiency. Technical efficiency is the ability of a business to gain a maximum output from the specific input. In contrast, allocative efficiency refers to the directing of resources toward products or services with the highest demand. Economic efficiency is allocative efficiency and technical efficiency from a joint unit of cost efficiency. An organization has an economic efficiency Which be efficient in terms of both technical and allocational [8]. In general, different methods have been used to measure hospital efficiency: Data Envelopment Analysis (DEA), Stochastic Frontier Analysis (SFA), and measures of performance, such as Pabon Lasso’s model. DEA is a non-parametric linear programming method used to evaluate the efficiency of decision-making units [8, 9]. SFA is parametric and calculates the difference between the organization’s predicted and expected outputs [10]. Pabon Lasso’s model (1986) assesses hospital performance using three performance indicators: bed occupancy rate (BOR), bed turnover rate (BTR), and average length of stay (ALS) [11].

A decline in hospital efficiency has been observed worldwide. In a global report by the World Health Organization (WHO) published in 2010, 10 sources of hospital inefficiency were identified: (1) underuse or overpricing of generic drugs; (2) use of substandard or counterfeit drugs; (3) inappropriate and ineffective drug use; (4) overuse or oversupply of equipment, investigations and procedures; (5) inappropriate or costly workforce mix, unmotivated worker; (6) inappropriate hospital admissions or length of stay; (7) inappropriate hospital size (low use of infrastructure); (8) medical errors and suboptimal quality of care; (9) waste, corruption and fraud; and (10) inefficient mix or inappropriate level of strategies [12]. However, thus far there has not been a comprehensive review to assess the source of hospital inefficiency in the EMR. This study aims to comprehensively identify the sources of hospital inefficiency in the EMR, and compare these to previously identified sources of hospital inefficiency. This will provide insight into the current condition of healthcare in this region.

According to the aforementioned WHO report, hospital efficiency in the EMR is low, particularly in low and middle-income countries (LMICs) [5]. To increase hospital efficiency in a context of rising costs and limited resources, it is necessary to identify sources of inefficiency and to suggest improvement strategies. Identifying these sources and identifying improvements are the objectives of this study.

## Methods

This is a systematic review of existing evidence on hospital inefficiency in the EMR. This study recruited English peer-reviewed articles published between January 1999 and May 2018. To identify relevant articles, a database search was conducted in MEDLINE (via PubMed) (Additional file 1), Web of Knowledge, Embase, Google and Google Scholar. Keywords used included “efficiency”, “productivity”, “inefficiency”, “hospital”, “data envelopment analysis”, “Pabon Lasso”, and “stochastic frontier analysis”. Moreover, the reference lists of selected articles were searched for relevant papers. Economic journals in the field of health economy and efficiency such as the Journal of the Knowledge Economy, the American Journal of Economics and Business Administration, Cost Effectiveness and Resource Allocation, and the International Journal of Economics and Financial Issues were searched individually. An initial review was conducted to determine the scope of the study, and no study published before 1999 was found. Therefore, the review included studies between 1999 and May 2018.

**Fig. 1:**
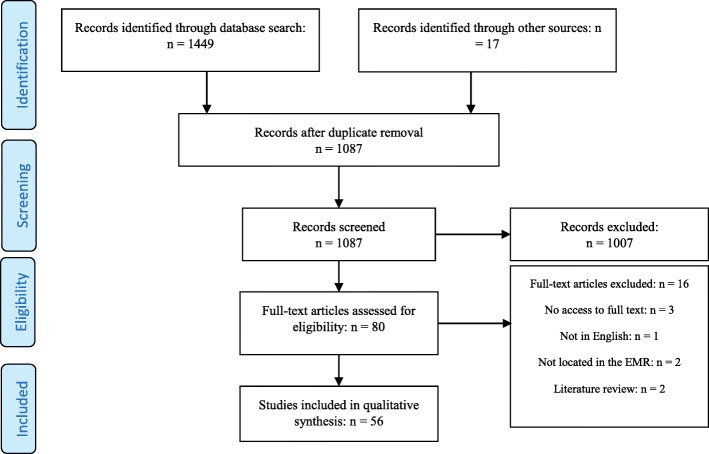
PRISMA Flow Diagram: Database search and article selection process

Following the screening of 1087 identified articles, 80 full texts were assessed for eligibility. After assessing these articles, 56 were included in the review. The screening process and search results are shown in the PRISMA Flow Diagram [13] of Fig. 1.

A data extraction form with entries for the first author, year of publication, country of study, data collection method, number of hospitals studied, inputs and outputs for efficiency, sources of hospital inefficiency, and factors affecting efficiency, was used to collect data from the selected studies. For higher reliability, two researchers independently extracted data from a randomly selected sample of the chosen articles. Any disagreements were solved by discussion and consensus and, if necessary, by a third reviewer.

Mitton et al.’s fifteen-point scale [14] was used for quality appraisal. The criteria used to assess quality included: literature review and identification of research gaps; research question and design, validity and reliability; data collection; population and sampling; and analysis and reporting of results. These criteria were rated 0 (not present or reported), 1 (present but low quality), 2 (present and mid-range quality), or 3 (present and high quality). Articles were rated independently by two researchers using the article quality rating sheet. Given that the review was qualitative, articles were not removed at this stage, but more weight was given to articles with a quality rating of 10 or above in the data analysis and interpretation of results.

The data were analyzed using qualitative content analysis. Data were coded and managed using MAXQDA 10 for Windows (VERBI GmbH, Berlin, Germany), and themes and subthemes were extracted to identify patterns and relationships between themes.

## Results

A total of 56 articles on hospital efficiency in the EMR, published between January 1999 and May 2018, were reviewed. A large number of studies (91%) were published after 2010. The reviewed studies were only conducted in 10 out of 22 EMR countries included in the search. Iran (*n* = 35) was most represented in the included studies, followed by Saudi Arabia (*n* = 5) and Tunisia (n = 5), Jordan (*n* = 4), Pakistan (*n* = 2), and finally UAE, Palestine, Iraq, Oman, and Afghanistan (*n* = 1 each).

Overall, 1995 hospitals were examined in these studies; most of them located in Iran (*n* = 858), Saudi Arabia (*n* = 573), Tunisia (*n* = 266), UAE (*n* = 96), Jordan (*n* = 72) and Afghanistan (*n* = 68). Out of 56 reviewed studies, 21 used DEA (37%), 12 used Bayesian SFA (21%), 10 used Pabon Lasso’s model (18%), and four studies used the Malmquist index (7.5%). Moreover, four studies (7.5%) used a hybrid approach by comparing DEA and Pabon Lasso’s model. Finally, five studies (9%) used other methods (the Cobb-Douglas Model, the Lean model, and efficiency and performance indicators).

**Fig. 2:**
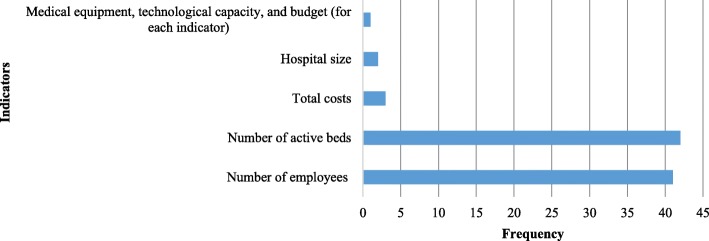
Frequency of input variables used to measure hospital efficiency in EMR countries

**Fig. 3:**
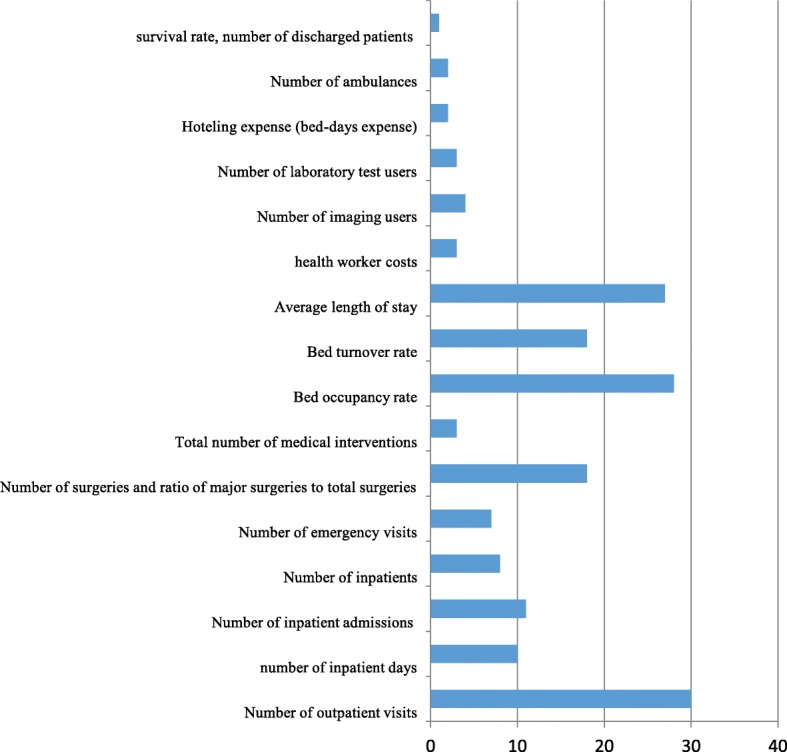
Frequency of output variables used to measure hospital efficiency in EMR countries

Calculating efficiency requires input and output variables. In data analysis, the number of workforce, active beds, total costs, hospital size, medical equipment, technological capacity, and budget have been used as input variables (Fig. 2). Total outpatient visits, inpatient admissions and days, number of inpatients, emergency visits, number of surgeries, ratio of major surgeries to total surgeries, total number of medical interventions, BOR, BTR, average length of stay (ALS), number of ambulances, ratio of active beds to fixed beds, hoteling expense (bed-day costs) and employee expense total survival rate, number of discharged patients, number of imaging service users, and number of laboratory test users, were used as output variables (Fig. 3). The input and output selection depends on the objective of the study and efficiency measurement. It is reasonable to consider total costs on the input side; however, few studies have employed hospital hoteling and workforce expenses as output in their evaluation. For example, Hatam [15] used hoteling and workforce expenses and found that most cases had more workforce and hoteling expenses than the similar ones showing significant inefficiency.

Operational definitions for acronyms and terms of input and output measures are given below:

- Number of active beds: alternative term for ‘available beds’ [16].
- Number of beds or hospital size: “Hospital beds include all beds that are regularly maintained and staffed and are immediately available for use. They include beds in general hospitals, mental health, and substance abuse hospitals, and other specialty hospitals. Beds in nursing and residential care facilities are excluded” [17].
- Number of inpatient admissions: Mean number of hospital admissions in a certain hospital per year [16].
- Number of bed-days: “number of days during which a person is confined to a bed and in which the patient stays overnight in a hospital” [18].
- Bed occupancy rate (BOR): “The occupancy rate for curative (acute) care beds is calculated as the number of hospital bed-days related to curative care divided by the number of available curative care beds, multiplied by 365”.
- Bed turnover rate (BTR): the number of times there is change of occupant for a bed during a given time period [17].
- Average length of stay (ALS): “Average length of stay refers to the average number of days that patients spend in hospital. It is generally measured by dividing the total number of days stayed by all inpatients during a year by the number of admissions or discharges. Day cases are excluded” [17].
- Day surgery: Day surgery is defined as the release of a patient who was admitted to a hospital for a planned surgical procedure and was discharged the same day [16].

Table 1 provides a summary of the studies reviewed, presenting the type and total number of hospitals examined, the methods used to calculate efficiency, inputs and outputs, and the source of inefficiency.

**Table 1:** Summary of reviewed studies

Author	Year	Country	Hospital type	Number of hospitals	Method used to calculate efficiency	Input and outputs	Source of inefficiency
Al-Shammari [19]	1999	Jordan	Hospitals of MoH*	15	DEA	Inputs: Numbers of bed-days, physicians, health workforce Outputs: Numbers of inpatient days, minor operations, major operations	Excess resources
Ramanathan [20]	2005	Oman	Regional and Wilayat hospitals (MoH), Sultan Qaboos University Hospital, Hospital of the Royal Oman Police	20	DEA (Malmquist index)	Inputs: Numbers of beds, physicians, and other medical workforces. Outputs: Number of visits, in-patient services, surgical operations	Partial utilization of inputs, lack of full compliance with technological changes
Hajialiafzali [21]	2007	Iran	Hospitals affiliated with the Social Security Organization	53	DEA (frontier-based methods)	Inputs: Total numbers of FTE* medical doctors, of FTE nurses, of other FTE workforces, number of beds Outputs: Numbers of outpatient visits and emergency visits, ratio of major surgeries to total surgeries, total numbers of medical interventions and surgical procedures	Partial utilization of inputs
Hatam [15]	2008	Iran	Hospitals affiliated with the Social Security Organization	18	DEA (frontier-based methods)	Inputs: Numbers of beds, FTE, total expense Outputs: Patient-days, BOR*, BTR,* ALS*, ratio of available beds to constructed beds, hoteling expense, bed-day costs, workforce costs	Unused beds
Goshtasebi [22]	2009	Iran	MoH hospitals	6	Pabon Lasso	Output: ALS, BOR, BTR	Underutilization of resources, high BOR
Jandaghi [23]	2010	Iran	Public and private hospitals	8	DEA (frontier-based methods)	Inputs: Numbers of physicians, nurses, medical workforce, official workforce, annual costs of hospital Outputs: Numbers of clinical visits, emergency visits, and bed-days	Excess resources
Hatam [24]	2010	Iran	General public hospitals	21	DEA (frontier-based methods)	Inputs: Numbers of hospital beds, FTE physicians, nurses, and other workforces Outputs: BOR, patient–day admissions, bed-days, ALS, BTR	Lack of motivation to select inputs to minimize expenses caused by the fact that hospitals are public and therefore do not seek profitability.
Shahhoseini [25]	2011	Iran	Provincial hospitals	12	DEA (frontier-based methods)	Inputs: Numbers of active beds, nurses, physicians, and other professionals Outputs: Number of surgeries, outpatients visits, BOR, ALS, inpatient days	Excess resources
Ketabi [26]	2011	Iran	Hospitals in Isfahan	23	DEA	Inputs: Average numbers of active beds, medical equipment, workforce (such as doctors, nurses and technicians) Outputs: BOR (%), ALS, total percentage of survival, performance ratio	Excess medical equipment, workforce and technology for teaching and private hospitals. Teaching hospitals are less efficient because of bureaucratic processes and private hospitals have lower BORs.
Bahadori [27]	2011	Iran	Hospitals affiliated with Urmia University of Medical Sciences	23	Pabon Lasso	Output: ALS, BOR, BTR	Poor performance in BOR and/or BTR in 60.87% of hospitals.
Al-Shayea [28]	2011	Saudi Arabia	Khalid University Hospital	1 (9 departments)	DEA	Inputs: doctors’ total salary, nurses’ total salary Outputs: Numbers of in-patients, outpatients, bed and average turnover rate	High costs of inputs
Kiadaliri [29]	2011	Iran	General hospitals affiliated with Ahvaz Jondishapour University of Medical Sciences	19	DEA (frontier-based methods)	Inputs: beds, human resources Outputs: inpatient days, outpatient days, number of surgeries, BOR	Inappropriate hospital sizes
Osmani [30]	2012	Afghanistan	District Hospitals	68	DEA and Tobit regression analysis model	Inputs: Numbers of physicians, midwives, nurses, non-medical workforce, and beds Outputs: Numbers of outpatient visits, inpatient admissions, and patient days, ALS, BOR, number of hospital beds (proxy for hospital size), bed-physician and outpatient physician ratio, number of physicians	Excess numbers of doctors, nurses, and beds
Farzianpour [31]	2012	Iran	Teaching hospitals of Tehran University of Medical Sciences	16	DEA (frontier-based methods)	Inputs: Numbers of physicians, practicing nurses in health facilities, and active beds Outputs: Numbers of inpatients, outpatients, ALS	Excess inputs or insufficient outputs
Chaabouni [32]	2012	Tunisia	Public hospitals	10	DEA and The Bootstrap Approach	Inputs: Numbers of physicians, nurses, dentists and pharmacists, other workforces, and beds Outputs: Numbers of outpatient visits, admissions, post-admission days	High hospital expenditures
Barati Marnani [33]	2012	Iran	Affiliated with Shahid Beheshti University of Medical Sciences	23	Pabon Lasso model and DEA (frontier-based methods)	Pabon Lasso: ALS, BOR, BTR DEA: Inputs: Numbers of physicians, nurses, other workforces, and active beds Outputs: BOR, numbers of patients and surgeries	Excess resources
Sheikhzadeh [34]	2012	Iran	Elected public and private hospitals of East Azerbaijani Province	6	DEA (frontier-based methods)	Inputs: Numbers of specialist physicians, general physicians, nurses, residents, medical team workforce with a degree (Bachelor’s), medical team, nonmedical and support workforce, and active beds Outputs: Numbers of emergency patients, outpatients, and inpatients, average daily inpatients residing in hospital	Excess and inefficient inputs: lack of medical services for the amount of resources used.
Yusefzadeh [35]	2013	Iran	Public hospitals	23	DEA	Inputs: Numbers of active beds, doctors, and other workforces Outputs: Number of outpatients’ admissions and day-beds	Excess inputs or insufficient outputs
Gholipour [36]	2013	Iran	Obstetrics and gynaecology teaching hospitals	2	Pabon Lasso	Output: ALS, BOR, BTR	Low BOR
Arfa [37]	2013	Tunisia	Public hospitals	101	DEA	Five fixed inputs: Numbers of physicians, dentists, mid-wives, nurses or equivalents, and beds. One variable input: budget Outputs: Numbers of outpatient visits and admissions	Hospitals are not operating at full capacity
Ajlouni [38]	2013	Jordan	Public hospitals	15	DEA and Pabon-Lasso	Pabon Lasso: ALS, BOR, BTR DEA: Inputs: Numbers of bed-days, physicians per year, and health workforce per year Outputs: Patient days, numbers of minor operations and major operations	Poor management, treatment of diseases requiring long patient stays
Abou El-Seoud [39]	2013	Saudi Arabia	Public hospitals that have been reformed to operate under private sector management through the full operating system in Saudi Arabia	20	DEA	Inputs: Numbers of specialists, nurses, allied workforce, and beds Outputs: Numbers of visits, patient hospital admissions, laboratory tests, and beneficiaries of radiological imaging	Administrative weakness to overcome external environmental factors rather than inability to manage internal operations
Bastani [40]	2013	Iran	Hospitals affiliated to the MoH	139	Four hospital performance indicators	Output: ALS, BOR, BTR	Inappropriate hospital sizes
Younsi [41]	2014	Tunisia	30 public and 10 private hospitals	40	Pabon Lasso	Output: ALS, BOR, BTR	Low bed density which may not match population hospital needs. Hospital bed numbers should be increased or maintained.
Torabipour [42]	2014	Iran	Teaching and non-teaching hospitals of Ahvaz County	12	DEA (Malemquist index)	Inputs: Numbers of nurses, beds, and physicians. Outputs: Numbers of outpatients and inpatients, ALS, number of major operations	Lack of familiarity of managers with advanced hospital technologies, lack of equipment and inappropriate use of technology in diagnosis, care and treatment.
Syed Aziz Rasool [43]	2014	Pakistan	Non-profit private organization (branches of LRBT hospitals)	16	DEA	Inputs: Numbers of beds, specialists, nurses Outputs: Numbers of outpatient visits, inpatient admissions, and total numbers of surgeries	Lack of government funds to hospitals run by non-profit organizations.
Pourmohammadi [44]	2014	Iran	All hospitals affiliated with the Social Security Organization	64	The Cobb-Douglas model	Inputs: Numbers of physicians, nurses, other workforces, and active beds Outputs: Number of outpatients and inpatients	Excess workforce
Mehrtak [45]	2014	Iran	All general hospitals located in Iranian Eastern Azerbijan Province	18	Pabon Lasso and DEA	Pabon Lasso: ALS, BOR, BTR DEA: Inputs: Numbers of active beds, physicians, nurses, discharged patients Outputs: Number of surgeries and discharged patients, BOR	Excess inputs: larger hospitals are more efficient than smaller hospitals.
Lotfi [46]	2014	Iran	All hospitals of Ahvaz (8 hospitals affiliated with Jundishapur University of Medical Sciences and 8 non-affiliated hospitals)	16	Pabon Lasso and DEA	Pabon Lasso: ALS, BOR, BTR DEA: Inputs: Numbers of physicians, nurses, other workforces, and active beds Outputs: BOR, numbers of patients and surgeries	Underuse of resources, excess hospital inputs
Kalhor [47]	2014	Iran	Hospitals affiliated with Qazvin University	6	Pabon Lasso	Output: ALS, BOR, BTR	Poor managerial decisions
Goudarzi [48]	2014	Iran	Teaching hospitals affiliated with Tehran University of Medical Sciences	12	DEA (frontier-based methods)	Inputs: Numbers of medical doctors, nurses, and other workforces, active beds, and outpatient admissions Outputs: Number of inpatient admissions	Excess numbers of nurses and active beds
Askari [49]	2014	Iran	Hospitals affiliated with Yazd University of Medical Sciences	13	DEA	Inputs: Numbers of active beds, nurses, physicians, and non-clinical workforce Outputs: hospitalization admissions, BOR (%), and number of surgeries	High excess inputs, particularly the excess number of nurses.
Adham [50]	2014	Iran	Teaching and non-teaching hospitals	14	Pabon Lasso	Output: ALS, BOR, BTR	Low BOR
Imamgholi [51]	2014	Iran	Hospitals affiliated to Busheher University of Medical Sciences	7	Pabon Lasso	Output: ALS, BOR, BTR	Non-optimal hospital sizes
Shetabi [52]	2015	Iran	Hospitals affiliated to Kermanshah University of Medical Sciences	7	DEA	Inputs: Numbers of active beds, doctors, nurses, and other workforces Outputs: Numbers of accepted inpatients, outpatients and BOR (%)	Excess inputs
Masoompourb [53]	2015	Iran	Teaching Hospital	1	Pabon Lasso	ALS, BOR, BTR	Decrease in ALS
Chaabouni [54]	2016	Tunisia	Public Hospitals	10	DEA (frontier-based methods)	Inputs: Numbers of physicians, nurses, dentists, pharmacists, and beds, total cost Outputs: Numbers of outpatient visits, admissions, and post-admission days, price of labor	large hospital sizes
Safdar [55]	2016	Pakistan	A large public hospital	1	DEA	Inputs: Waiting time at the pharmacy, length of waiting line Outputs: Consultation time at the pharmacy	High waiting times: low efficiency levels (less than 50% efficiency) are associated with high waiting times.
Mohammadi [56]	2016	Iran	Public hospitals	67	Cobb-Douglas production function	Inputs: Human resources (including net working hours of specialized workforce) and bed numbers (including the number of active beds)	Insufficient inputs: Inpatient service production levels were lower than expected in 40% of hospitals. A 10% increase in net working hours of specialized human resources would generate a 8.8% increase in average inpatient service production levels. A 10% increase in the number of active beds would generate a 1.1% increase in average inpatient service production levels.
Mahate [57]	2016	United Arab Emirates	Private and public hospitals in the UAE	96	DEA	Inputs: Numbers of beds, doctors, dentists, nurses, pharmacists and allied health workforce, and administrative workforce Outputs: Numbers of treated inpatients, outpatients, ALS	Waste of 41 to 52% of inputs during service delivery.
Kalhor [58]	2016	Iran	Tehran city general hospitals	54	DEA	Inputs: Total numbers of FTE medical doctors, and nurses, numbers of supporting medical workforce including ancillary service workforce, and beds Outputs: Numbers of patient days, outpatient visits, patients receiving surgery, ALS	Ownership type (lower efficiency of university hospitals because of more expenditures)
Kakemam [59]	2016	Iran	Hospitals of public, private, or social security ownership types in Tehran	54	DEA	Inputs: Numbers of active beds, physicians, nurses, and other medical workforces Outputs: Numbers of outpatient visits, surgeries, and hospitalized days, ALS	Lack of resource optimization. Poor adaptation of the sizes, types of practices, and ownerships of hospitals, affecting their technical efficiency. Approximately 70% of the hospitals were inefficient.
Hassanain [60]	2016	Saudi Arabia	Hospitals affiliated to the MoH	12	Lean	On-time start, room turnover times, percent of overrun cases, average weekly procedure volume and OR utilization	Ppoor hospital infrastructure, old technology, suboptimal management of human resources, the absence of employee engagement, frequent scheduling changes, inefficient process flow
Hamidi [61]	2016	Palestine	22 government hospitals	22	DEA (frontier-based methods)	Inputs: Numbers of beds, doctors, nurses, and non-medical workforce Outputs: Numbers of admitted patients, hospital days, operations, outpatient visits, ALS	Mismanagement of available resources, shortage of the numbers of doctors and nurses and excess number of non-medical staff
Nabilou [62]	2016	Iran	Hospitals affiliated to Tehran University of Medical Sciences	17	DEA (Malmquist index)	Inputs: Active beds, nurses, doctors and other workforces Outputs: outpatient admissions, bed-days, number of surgical operations	Due to hospitals’ technological changes, a lack of knowledge of hospital workforce on proper applications of technology for patient treatment became the main cause of low hospital productivity and inefficiency.
Rezaei [63]	2016	Iran	Kurdistan teaching hospitals	12	DEA (frontier-based methods)	Inputs: Numbers of active beds, nurses, physicians, and other workforce members Outputs: Inpatient admissions	Waste of inputs during service delivery
Farzianpour [64]	2017	Iran	Training and non-training hospitals of Tabriz city	19	DEA	Inputs: Numbers of physicians, total workforce, and active beds Outputs: Number of outpatients and BOR	Poor management of human and financial resources.
Arfa [65]	2017	Tunisia	Public district hospitals	105	DEA	Inputs: Numbers of physicians, surgical dentists, midwives, nurses and equivalents, and beds, operating budget Outputs: Outpatient visits in stomatology wards, outpatient visits in emergency wards, outpatient visits in external wards, numbers of admissions, and admissions in maternity wards	Inadequate number of workforce, equipment, beds, and medical supply, health quality and lack of fitting operating budgets: tackling these sources of inefficiency would reduce net user needs and the bypassing of the public district hospitals, to increase their capacity utilization. Social health insurance should be turned into a direct purchaser of curative and preventive care for the public hospitals.
Aly Helal [66]	2017	Saudi Arabia	Public hospitals	270	DEA	Inputs: Numbers of beds, doctors, nurses, and allied medical workforce Outputs: Numbers of individuals visiting admitted patients, radiography service beneficiaries, laboratory testing beneficiaries, and inpatients	Excess inputs
Mousa [67]	2017	Saudi Arabia	Public hospitals	270	DEA	Inputs: Numbers of physicians, nurses, pharmacists, allied health professionals, beds Outputs: Numbers of outpatient visits, inpatients, laboratory investigations, X-rays patients, X-rays films, total number of surgical operations	Inadequate resources: some resources should be switched between regions to improve efficiency.
Moradi [68]	2017	Iran	Public hospitals	11	Pabon Lasso	ALS, BOR, BTR	Low number of hospital beds, and need for hospital expansion
Sultan [69]	2017	Jordan	General public hospitals	27	DEA	Inputs: Numbers of beds, physicians, healthcare workforce, administrative workforce Outputs: Inpatient days, outpatient visits, emergency departments, and ambulances	Diseconomies of scale affect the operational efficiency, poor management, poor productivity in outpatient services and low numbers of physicians.
Kassam [70]	2017	Iraq	Hospitals in Baghdad	3	DEA and Luenberger Productivity Indicator (LPI)	Inputs: Numbers of doctors, nurses, and other health workforces Outputs: Numbers of outpatients, laboratory tests, radiology tests, sonar tests, emergency visits	The cause of the inefficiencies is undetermined.
Rezaee [71]	2018	Iran	Hospitals affiliated with Kermanshah University of Medical Sciences	15	Pabon Lasso	Output: ALS, BOR, BTR	Excess inputs
Yazan Khalid Abed-Allah Migdadi [72]	2018	Jordan	Public hospitals	15	DEA	Inputs: Numbers of physicians, nurses, and beds Outputs: ALS, number of Surgeries, BOR	Low BOR
Sajadi [73]	2018	Iran	All hospitals in Isfahan City	54	Cross-sectional descriptive study comparing performance indicators	Outputs: BOR, BTR, bed-days, inpatients visits, number of surgeries in all types of hospitals, outpatient visits in all non-private hospitals, emergency visits in public and social security hospitals, and natural deliveries in public and semi-public hospitals	Inefficient use of limited resources

*BOR bed occupancy rate, *BTR* bed turnover rate, *ALS* average length of stay, *FTE* Full Time Employee, *MoH* Ministry of Health

Various sources of hospital inefficiency were identified and divided into four themes, each with a set of subthemes: hospital products and services, hospital workforce, hospital services delivery, hospital system leakage (Table 2).

**Table 2:** Source of inefficiency in Eastern Mediterranean hospitals and strategies for improvement

Source of inefficiency		Common sources of inefficient performance	Proposed actions
Hospital products and services	overuse or supply of equipment, investigations, and procedures	- Inappropriate payment systems (fee-for-service payment mechanisms) - Misuse or inappropriate use of technology in patient treatment and diagnosis like imaging and lab services due to lack of knowledge and skills of health professional and lack of adopted evidenced-based guidelines. - Overuse or oversupply of equipment - Lack of or defective hospital equipment - Poor standards for use of technologies	-Reform incentive and payment structures, developing appropriate tariff and payment systems (e.g. use capitation or diagnosis-related group mechanism for reimbursement) -Raising workforce awareness and training workforce and managers about new information systems and technologies -Raising workforce awareness of energy management through frequent training -Develop and implement clinical guidelines
Hospital workforce	inappropriate or costly workforce mix	- Lack of or failure to use specialized managers in hospital administration - Suboptimal use of workforce capabilities, including those of physicians, nurses, paramedics, and support workforce, resulting in excess workforce in some departments - Inadequate management of hospital resources like workforce	-Recruiting workforce based on hospital needs (both in terms of numbers and specialties required) -Preventing the recruitment and maintenance of specialist workforce who are not significantly relevant to hospital and patient needs. -Using work measurement and time management techniques for optimal use of the workforce with respect to the volume of hospital operations
	unmotivated workforce	- Lack of motivation due to high workload - Lack of workforce motivation in the public sector because of inadequate salaries	-Introducing performance-based payments -Use appropriate incentive, reward and appraisal systems
Hospital services delivery	inappropriate hospital admissions and length of stay	- Inappropriate ALS*, unnecessary admissions, low BORs* and unnecessary referrals to specialists due to inadequate knowledge and training of workforce about best practice.	-Developing and implementing policies to accelerate admission and discharge processes and increase the quality of services -Developing strategies to reduce ALS*, including full-time presence of physicians and modification of hospital funding policies -Establishing a two-way electronic referral system, to provide physicians with feedback -Effective marketing using appropriate customer information, and improving communication and customer loyalty
	inappropriate hospital size (low use of infrastructure)	- Inefficient hospital size, lack of scale efficiency and too many hospitals and inpatient beds in some areas, not enough in others - Suboptimal use of available capacities such as infrastructure and active beds, resulting in excess beds in some departments (lack of planning)	-Modifying hospital size: selecting an efficient size and preventing hospital overdevelopment. if inefficient (downsizing or merging hospitals) -Making optimal use of hospital beds based on community needs. -Use of cost analysis and DEA model and other efficiency measurement models for incorporate inputs and output estimation into hospital planning. -Improving workforce, equipment, and beds based on evidence -Designing a basic framework for optimal resource allocation by health policymakers -Diversifying the outputs required for compensating hospital inefficiency -Redistributing hospital resources among regions -Training to raise knowledge about efficient admission practice
	medical errors and suboptimal quality of care	- Poor care management skills of physicians and other workforces. - Inadequate managerial skills and lack of training for hospital managers. - Inadequate skills and training of the hospital workforce.	-Designing on-the-job training courses tailored to workforce roles. -Using experienced and well-educated managers with management or healthcare management degrees, performance evaluation of hospital managers and provide feedback -Introducing managers to management techniques and methods of economic analysis -Improve hygiene standards in hospitals; provide more continuity of care; undertake more clinical audits; monitor hospital performance
Hospital system leakages	waste, corruption and fraud	- Inappropriate suboptimal allocation of funds among hospitals and unclear resource allocation guidance. - Hospital reliance on public funds and budgets, and lack of competition with other organizations.	-Modifying hospital budget structures -Improve regulation/governance, including strong sanction mechanisms; assess transparency/vulnerability to corruption; undertake public spending tracking surveys; promote codes of conduct

*BOR bed occupancy rate, *BTR* bed turnover rate, *ALS* average length of stay

The most frequent sources of inefficiency in EMR hospitals are excess workforce, excess beds, and inappropriate hospital sizes. Helal et al. [66] investigated the effect of health reforms (privatization) on the efficiency of 270 hospitals in Saudi Arabia and reported a 0.90 average efficiency in 2006 and a 0.92 average efficiency in 2014. The average efficiency of one is considered the best level of performance. Despite a reduction in inputs, outputs increased by 2%. Moreover, there was a 10.1% increase in the number of inpatients from 2006 to 2014. Therefore, reducing excess inputs such as excess workforce, excess beds or/and increasing outputs can be beneficial to hospitals. A 2013 analysis in Saudi Arabia showed that there was a reduction in the number of beds, doctors, nurses, and allied health workforce as inputs. Moreover, there was an increase in the number of inpatients, outpatients, the number of daily laboratory tests and the number daily of radiography services as outputs [39]. The most common strategies proposed in the included studies are: developing health policies for accurate recruitment planning, calculating the required number of beds for each community, and making proper use of hospital beds based on community needs.

## Discussion

The purpose of this research was to examine the sources of hospital inefficiency and strategies available to increase hospital efficiency in the EMR. In recent years, there has been an increasing focus on hospital efficiency for health policymakers in developing countries. A total of 56 studies have been conducted on hospital efficiency in the EMR from January 1999 to May 2018. These studies have shown that hospital care is an economic activity requiring adequate funding and budgeting. As such, reducing inputs can improve performance and efficiency [56, 74].

The WHO Regional Office for the EMR classifies countries to there groups: high income countries (six countries), middle income countries (ten countries), and low income countries (six countries). The present research identified 56 articles on hospital efficiency in three high-income countries, five middle-income countries, and two low-income countries. General government expenditure allocated to health in the EMR countries remains between 2 and 16%, a low figure. Regarding hospital service utilization, the overall average bed occupancy rate and length of stays were 60.7% and 4.12 days, respectively, in the Region in 2013. Only a few countries have well-defined and functioning referral networks between hospitals and primary health care facilities, or between hospitals at different levels. Hospitals do not serve geographically defined catchment areas based on national policy mandates. Most countries are entrenched in the historical model of public provision and financing, and there is a mix of funding patterns, including public sector funds (through central government budgets and national insurance funds) and out-of-pocket payments made directly by users. In most countries, there is misalignment between the distribution of hospital beds and high-technology equipment and population health needs [4]. Contextual challenges exist, such as security issues, internal conflict and political volatility in EMR countries, leading to economic problems influencing health policies, health system budgets, and health system efficiency as a result [75, 76].

Some health system challenges are common to all EMR countries: “limited capacity in MoHs for evidence-based policy analysis and formulation and strategic planning through better use of information in adequate capacity to legislate, regulate and enforce rules and regulations” or “most countries lack national medicines policy” [75]. Both this study and the WHO have reported similar findings.

The most common input variables used in these studies were workforces numbers and the number of beds, while the most common output variables were the total number of outpatient visits, admissions and inpatient days. A systematic review of new approaches to measure hospital performance in LMICs in 2015 [77] identified seven key performance indicators. These included total inpatient days; recurrent expenditure per inpatient day; ALS; infection prevention rate; BOR; inpatient days per technical workforce; and unit cost of outpatient care. Seven performance indicators were also identified for high-income countries (HICs): mortality rate from emergency heart attack admissions after 28 days; mortality rate from emergency surgery after 30 days; number of patients on waiting lists; infection rate of methicillin-resistant *Staphylococcus aureus* per 10,000 bed-days; net profit; probability of workforce leaving within 12 months; and average healthcare commission rating [77].

On average, out-of-pocket payments differ between HICs and LMICs. In HICs, patients rarely pay directly for their care compared to LMICs where direct payment by patients is necessary due to lower insurance coverage. Furthermore, the mortality rate for non-elective admission is not the optimal output indicator for LMICs, as access to healthcare is a significant problem. These explain the differences in outputs between LMICs and HICs [77, 78].

The themes related to inefficiency extracted in this review, and the sources of inefficiency identified in the WHO report 2010 [11], highlight that studies have failed to address the issue of medical drugs. Using drug-related inputs and outputs can provide useful insights into drug-related sources of inefficiency in the EMR. For example, a study in Ethiopia used the cost of drug supply as input [79]. This can provide further insights into how to improve hospital efficiency.

In addition to excess workforce, excess beds and inappropriate hospital sizes, the inefficiency of hospitals in the EMR is also due to inappropriate workforce composition, lack of workforce motivation and inefficient use of health system inputs. According to a WHO report about National Health Accounts published in 2009, 15 to 25% of hospital inefficiency is related to workforce [80]. The workforce is at the core of the health system and accounts for almost half of the total health budget, in the form of wages and other payments [81]. The shortage of human resources is a major obstacle in implementing national healthcare plans, causing ineffective recruitment, inappropriate training, poor supervision, and suboptimal workforce distribution, which can further reduce efficiency [82]. Strategies to increase workforce efficiency focus on assessment and training based on needs, reviews of incentive policies, flexible contracts and performance-based payments [83].

Hospitals can result in lower efficiency if healthcare products and services are not optimal. Hospitals will face higher inputs against the specific output or lower outputs against the specific input. Excessive lengths of hospital stays, unnecessary admissions, and unnecessary referrals to specialists are examples of overuse of healthcare services. Reduced demand for hospital services and low BORs indicate underuse of available services [25–32]. A WHO report showed that suboptimal use of hospital resources, such as doctors, nurses, and beds, reduce demand for services and thus reduce hospital efficiency [82]. Optimal hospital management plays a vital role in optimizing healthcare services, improving hospital outcomes, and reducing costs [84–86]. Hospital managers and health policymakers can increase hospital efficiency and productivity through economies of scale. Strategies include optimizing hospital size, providing more products and services, and reducing ALS [38, 84–86].

Two of the principal sources of inefficiency in the EMR are inappropriate hospital sizes and excess numbers of active beds. These have been analyzed in studies conducted in countries outside the EMR, including in HICs [14, 21, 24–26, 33–35, 62]. These studies revealed the significant impact of hospital size and bed numbers on efficiency [87, 88]. The optimal number of active hospital beds typically lies between 200 and 300 beds. Generally, hospitals with less than 200 beds or more than 600 beds have higher costs [89]. According to international standards, a threshold BOR range between 84 and 85% indicates that use of hospital facilities and hospital resources are optimally efficient [90]. Therefore, optimizing hospital sizes and bed numbers can ensure that hospitals respond to population needs thus increasing efficiency. Indeed, it may be necessary for governments to build hospitals of a specific size, to take into account geographical considerations and difficulties accessing healthcare facilities.

The payment system has a vital role in improving hospital efficiency and productivity. In the EMR, payment systems are typically fee-for-service systems. In developed countries payments are often based on performance at clinical and organizational levels, increasing efficiency through performance incentives [91, 92]. Strategies to increase hospital efficiency include developing healthcare policies to implement appropriate payment systems, fair tariffs, and meticulous workforce recruitment plans, calculating required bed numbers for each community, making optimal use of hospital beds based on demand, and developing two-way electronic referral systems.

## Conclusion

The results of this study have elucidated numerous sources of hospital inefficiency in the EMR. These sources should be addressed with targeted strategies, to improve hospital performance. Severe resource scarcity and increased costs of healthcare services, particularly in developing countries, require policymakers to ensure maximum use of available resources. Hospitals are highly complex, multidisciplinary social entities, whose performance can be improved through accurate, effective, and timely planning, organization, leadership, and management. Efficiency depends on multiple factors. As such, using various methods to measure hospital efficiency can be an effective strategy for managers and policymakers. Needs-based assessments and training, reviews of incentive policies, flexible contracts, performance-based payments, optimal hospital sizes based on community needs, increased resource availability and preservation of hospital social functions are crucial to increasing hospital efficiency.

## Supplementary information

**Additional file 1.** Search strategy in Medline via PubMed.

## Data Availability

Not applicable.

## Abbreviations

ALS: Average length of stay
BOR: Bed occupancy rate
BTR: Bed turnover rate
DEA: Data Envelopment Analysis
EMR: Eastern Mediterranean Region
FTE: Full Time Employee
HICs: High-income countries
LMICs: Low- and middle-income countries
MoH: Ministry of Health
SFA: Stochastic Frontier Analysis
WHO: World Health Organization
