# Correction to: A systematic review on hospital inefficiency in the Eastern Mediterranean Region: sources and solutions

**DOI:** 10.1186/s12913-019-4766-x

**Published:** 2019-12-10

**Authors:** Hamid Ravaghi, Mahnaz Afshari, Parvaneh Isfahani, Victoria D. Bélorgeot

**Affiliations:** 1 Department of Health Service Management, School of Health Management and Information Sciences, Iran University of Medical Sciences, Tehran, Iran; 2 School of Public Health, Zabol University of Medical Sciences, Zabol, Iran; 3 World Health Organization, Regional Office for the Eastern Mediterranean, Monazamet El Seha El Alamia Street, Extension of Abdel Razak El Sanhouri Street, Nasr City, Cairo, Egypt

**Correction to: BMC Health Serv Res (2019) 19:830**

**https://doi.org/10.1186/s12913-019-4701-1**

In the original publication of this article [1], one author’s name needs to be revised from Pavaneh Isfahani to Parvaneh Isfahani. The original article has been corrected.
