# Supplementary material for: A systematic review on hospital inefficiency in the Eastern Mediterranean Region: sources and solutions
Source: BMC Health Serv Res. 2019 Nov 12;19:830. doi: 10.1186/s12913-019-4701-1 (PMC6852759; doi:10.1186/s12913-019-4701-1)
Supplement: Supplementary file 1 — Additional file 1. Search strategy in Medline via PubMed. [file 12913_2019_4701_MOESM1_ESM.docx]

**Additional file 1: Search strategy in Medline via PubMed**

((((“Efficiency”[mesh] OR “Productivity”[mesh] OR “Organizational Efficiency”[tiab] OR “inefficiency”[tiab] OR “Productivity, Organizational”[tiab] OR “Organizational Productivity”[tiab] OR “Program Efficiency”[tiab] OR “Efficiency, Program”[tiab] OR “Efficiency, Administrative”[tiab] OR “Administrative Efficiency”[tiab] OR “Efficiency”[tiab] OR “Data Envelopment Analysis”[tiab] OR “Pabon Lasso”[tiab] OR “Stochastic Frontier Analysis”[tiab] OR “Productivity”[tiab])) AND (“Hospital”[mesh] OR “hospital”)) AND (“Afghanistan” OR “Bahrain” OR “Djibouti” OR “Egypt” OR “Iran (Islamic Republic of)” OR “Iraq” OR “Jordan” OR “Kuwait” OR “Lebanon” OR “Libya” OR “Morocco” OR “Oman” OR “Pakistan” OR “Qatar” OR “Saudi Arabia” OR “Somalia” OR “Sudan” OR “Syrian Arab Republic” OR “Tunisia” OR “United Arab Emirates” OR “Yemen” OR “Palestin”))
